# Semi-field studies on biochemical markers of honey bee workers (Apis mellifera) after exposure to pesticides and their mixtures

**DOI:** 10.1371/journal.pone.0309567

**Published:** 2025-01-30

**Authors:** Agnieszka Murawska, Ewelina Berbeć, Krzysztof Latarowski, Adam Roman, Paweł Migdał

**Affiliations:** 1 Department of Bees Breeding, Institute of Animal Husbandry and Breeding, Wroclaw University of Environmental and Life Sciences, Wroclaw, Poland; 2 Department of Human Nutrition, Wroclaw University of Environmental and Life Sciences, Wroclaw, Poland; University of British Columbia, CANADA

## Abstract

Due to the fact that many different pesticides are used in crop production and their residues can accumulate in the environment, bees are in contact with various pesticides at the same time. Most studies on their influence on honey bees focus on single substances in concentrations higher than those found in the environment. Our study assessed the chronic effects of commonly used pesticides and their mixtures on selected biochemical markers in worker bee hemolymph. Workers developed in the hive and were provisioned with to pesticides in concentrations corresponding to residues detected in pollen, honey, and/or nectar. Colonies were exposed daily to 0.5L for 7 days by feeding a sugar syrup containing a formulation of acetamiprid (250 ppb) (insecticide), glyphosate (7200 ppb) (herbicide), and tebuconazole (147 ppb) (fungicide) administered alone, in a binary or ternary mixture. Administered alone, acetamiprid significantly decreased the level of urea in the hemolymph of worker honey bees. Glyphosate did not affect significantly the level/activity of any of the biochemical markers. Tebuconazole caused changes in the levels of most of the studied biochemical markers. We found that tebuconazole, which as a fungicide is generally considered safe for bees, may be harmful and more research is required. The impact of fungicides is a crucial element of the assessment of threats to honey bees.

## Introduction

Among all the threats, especially those of anthropogenic origin, pesticides are mentioned as one of the main factors harmful to honey bees [1]. Honey bees can be poisoned with pesticides through contact and/or ingestion. Plant nectar and pollen accumulate systemic pesticides, which are commonly used in developed countries [2,3]. Among the many pesticides allowed around the world, widely used are glyphosate (herbicide), acetamiprid (insecticide) and tebuconazole (fungicide).

Glyphosate is now the most commonly used herbicide. It accounts for 71.6% of all pesticide active ingredients sold worldwide [4]. Due to the introduction of glyphosate-resistant crops in 1996, the herbicide’s active ingredient glyphosate has rapidly increased in use. This substance is also widely used in European agriculture, where glyphosate-resistant crops have not been adopted as part of common agricultural practices [5]. It is a herbicide belonging to the group of amino phosphonates. By inactivating the EPSP enzyme, it causes disruptions in the biosynthetic pathway of amino acids such as phenylalanine, tryptophan and tyrosine. This leads to amino acid starvation and, as a result, the death of the plant [6]. It was introduced to the market in 1975 by Monsanto Chemical Company under the name Roundup ^™^ [7]. It works systemically. It has been widely used for over 40 years, thanks to its wide spectrum of actions. Although glyphosate was initially considered safe for honey bees (as they do not have EPSP enzyme) more and more reports indicate its negative impact, even at the doses recommended by the manufacturer [8]. Bees in contact with plants sprayed with glyphosate (10,000 ppb and 20,000 ppb) had higher mortality than the control group [9]. The addition of glyphosate to the larval diet resulted in increased mortality (4,000 or 20,000 ppb) [10] and reduced weight and delayed molting (1,250–5,000 ppb) [11]. Glyphosate administered to bee colonies in syrup (4,800 ppb and 137,600 ppb) caused little impact on the condition of the colony and the survival of individual workers. The higher concentration did contribute to slower brood development, but this did not affect the general condition of the colonies [12]. However, in colonies fed with syrup with the addition of glyphosate at a concentration of 75,000, 150,000 or 301,000 ppb, no significant effect on survival, development, average brood weight or worker mortality was observed [13]. Wintering honey bees were fed food containing glyphosate, imidacloprid and difenoconazole, individually or in mixtures (0.1, 1 and 10 ppb) for 20 days. Bee mortality was higher after exposure to these 3 pesticides, both individually and in combination [14]. Moreover, Motta and Moran found that the microbiota of honey bees is affected by sublethal doses of glyphosate (0.04 to 1.0 mM) [15] which leads to immune dysregulation [16].

Acetamiprid is one of the most frequently determined plant protection products in honey [17]. It is a cyano-substituted insecticide from the neonicotinoids group, chemically related to nicotine. Acetamiprid works systemically. Like other neonicotinoids, it is an acetylcholine antagonist at postsynaptic membrane receptors in insect nerve cells. By binding to receptors instead of acetylcholine, it is not broken down by the enzyme acetylcholinesterase. This causes stimulation and disturbance in nerve impulse conduction [3]. Acetamiprid is used to control sucking and biting pests (mainly Hemiptera, Lepidoptera, and Thysanoptera), on rapeseed crops, in orchards, in the cultivation of vegetables and ornamental plants, as well as in forests [18]. Depending on the dose or concentration and the method of application, acetamiprid may increase honey bee mortality. This effect is mainly observed in laboratory studies [19–23]. After application on the thorax, acetamiprid at doses of 1,000 and 2,000 ng/bee increased workers’ mortality compared to the control group. A dose of 2,000 ng/bee shortened workers’ lifespan by approximately 4 days [19,24]. Bees fed with syrup with the addition of this insecticide at concentrations of 600, 1,200, 2,400, 6,000 and 60,000 ppb had mortality rates below 25% [21]. Workers fed with acetamiprid solutions in the amount of 25,000 ppb (from 2-day-old larvae to 14-day-old imago, except for the pupal stage) were characterized by a shorter lifespan than the control group by approximately 1 day, a lower weight after breaking out and a delay in breaking out of the cells. Exposure also resulted in a reduction in the number of mated brood cells [25].

Azole fungicides, including tebuconazole, are among the most commonly used fungicides against fungal diseases in agricultural crops [26]. Knowledge of how fungicides harm honey bees is still scarce. However, there is evidence that fungicides can increase the toxicity of insecticides [27,28]. Tebuconazole inhibits the synthesis of ergosterol, which is essential in the formation of the mycelium and fungal cell membrane. Tebuconazole acts systemically [26]. There are only a few studies on tebuconazole’s effects on the honey bee [27,29] and may impair energy production [30]. Tebuconazole (2,000 ng/bee) increased the toxicity of thiacloprid. Honey bees exposed to tebuconazole or thiacloprid did not show higher mortality than control bees, but the combination of these agents resulted in mortality in 70% of the bees tested [31]. In other studies, tebuconazole (447 ng/bee orally or by contact) in combination with neonicotinoids resulted in increased toxicity, the highest level of synergy, 2.6-fold, was observed between tebuconazole and thiamethoxam [32]. Tebuconazole can affect microorganisms in various environments, including the insect gut [33,34]. In a study of the transfer of tebuconazole residues from wax and then to royal jelly in cells where queen bees were developing, no tebuconazole residues were detected in queen larvae and newly emerged bees [35]. Tebuconazole is used for prophylactic purposes and to combat fungal diseases, e.g., in rapeseed and fruit crops. Tebuconazole is one of the most frequently determined fungicides in honey [17].

Pesticides can be co-applied in tank mixes, and even if they are not combined, honey bees flying on multiple crops and collecting nectar or water can be exposed to them in the field. Over 64% of all agricultural land (or around 2.5 billion ha) is at risk of contamination by more than one pesticide’s active substance. Honey bees are exposed to harmful substances not only in the environment, but also in the hive as they collect pollen and nectar that may be contaminated. [3]. Complex toxicological interactions can lead to unpredictable pesticide mixture results. When combined, there may be an additive effect (i.e., equal to the sum of the individual substances), a synergistic effect (greater than the additive effect), or an antagonistic effect (less than an additive effect) [22]. It can be especially dangerous when synergistic effects occur. In the synergistic mixtures identified so far, organophosphates, N-methylcarbamates, triazoles, triazine herbicides, and pyrethroid insecticides are present [36]. Our previous study showed that mixtures of acetamiprid and tebuconazole, acetamiprid and glyphosate as well as ternary mixture of these substances have synergistic effect on honey bee mortality [37].

The honey bee is constantly exposed to a mixture of different chemicals, usually in low concentrations, which can have long-term effects on its health [3]. Consequently, it is crucial to study the combined effects of different classes of pesticides at environmental concentrations. Residues of acetamiprid, tebuconazole and glyphosate are found in nectar, pollen, honey, wax and waterso they pose a potential threat to the honey bee [12,17,35,38–49].

Plant protection products can trigger detoxification mechanisms and antioxidant properties of the honey bee body [14,50–55], we decided to analyze the activity of detoxification and antioxidant enzymes–alanine aminotransferase (ALT), aspartate aminotransferase (AST), alkaline phosphatase (ALP) Gamma-glutamyl transpeptidase (GGTP) and the level of non-enzymatic antioxidants–creatinine, uric acid and urea, as well as an indicator of antioxidant capacity of organisms–total antioxidant status (TAS) in the hemolymph of worker bees. AST and ALT are involved in the metabolism of amino acids, and ALP catalyzes the dephosphorylation of many phosphate esters [56,57]. The presence of these enzymes has been detected in the hemolymph of many insects, including bees. ALP activity is also determined in honeybee intestinal tissues [14,55,58,59]. GGTP is an enzyme widely found in organisms, from bacteria to mammals [60]. It was marked, among others, in the hemolymph of honey bees and the wax moth (*Galleria mellonella* L.) [61,62]. GGTP plays a key role in the gamma-glutamyl cycle pathway 13 synthesis and degradation of glutathione and detoxification of xenobiotics, catalyzing the early stages of glutathione degradation and producing cysteinylglycine [63–66]. Cysteinylglycine is a compound thiol with very high reactivity and creates reactive oxygen species, which in turn facilitates the oxidative reactions [67,68]. Creatinine is a product of the breakdown of phosphocreatine found in muscle tissue. Urea has been detected in the hemolymph and excrement of many insects, including honey bees [57,69,70]. It is considered a secondary end product of nitrogen metabolism [71,72]. The end product of nitrogen metabolism is also acid urine [73]. This compound is determined in the hemolymph of honey bees [57,69,70,74]. The reduced activity of enzymes observed in the honey bee hemolymph due to adverse or harmful factors may mean that the release of AST, ALT, ALP and GGTP was suppressed (or that harmful factors blocked them, thus directly reducing their activity (inhibition or both) [75]. Low activity of these enzymes in bees may impair the Krebs cycle, ATP synthesis, oxidative phosphorylation, β-oxidation [76], and may also indicate the presence of insufficient amount of protein in the diet, which is necessary, among others, for tissue formation [77]. Changes observed in creatinine levels in the hemolymph may indicate increased muscle work or increased metabolism ([78]. Changes in the levels of uric acid and urea can be a sign of malnutrition or starvation. Uric acid, like urea, is an end product of nitrogen metabolism [71]. It has been proven that in some insects they can also be formed as end products of protein metabolism [79].

The study was semi-field. Workers developed in the hive and were exposed to pesticides in concentrations corresponding to residues detected in pollen, honey, and/or nectar. We decided to use commercial formulation as is recommended in semi-field and field studies with honey bees [80]. In commercial formulations, surfactants, stabilizing agents, dispersants, and sometimes synergists are added after dilution of the active substance [81], which can change toxicity for honey bees. This was done to create realistic scenarios. Our hypotheses assume that: 1) the active substances in the selected formulations and concentrations will have a different effect on the level or activity of selected biochemical indicators depending on the application (single or in mixtures), 2) the most biochemical markers will be affected by an insecticide.

## Materials and methods

### Worker bees

Worker bees were reared in 40 colonies of honey bees (Apis mellifera carnica). The experiment was carried out from July 2021 to August 2022. The apiary was located in Nowa Jablona, Lubuskie, Poland (51°37′44″N 15°48′37″E). There were no visible signs of diseases. The honey bees had not been treated with any medicinal treatments for 4 months prior to the experiment. At the beginning of the experiment each experimental colony consisted of 4 combs covered by bees including one comb with eggs, larvae, capped cells, and bee pollen without carbohydrate stores. Until the exposure time (i.e., 14 days), colonies were fed sugar cake Apikand (Lyson, Sulkowice, Poland) to provide carbohydrates. In each colony, an *Apis mellifera carnica* queen originating from the same mother-queen was inseminated with the multiple drones semen from the same father-queen colony. Two days before exposure to pesticides, an empty comb was placed in each colony so that the queen could lay eggs on it. Combs were marked with pin. Next day, combs were checked for the presence of eggs. 21 days after eggs laying, newly emerging workers from each group were collected immediately from the marked comb in the hive for hemolymph analysis (We collected about 300 bees from each group (around 60 bees from each hive). No permits approved for the field site access were required, because the field site is our private property and because bees were fed in-hive there was limited risk to the environment.

### Exposure to pesticides

Exposure took place in the period with limited plant blooming (i.e., July) for little risk of colonies being exposed to pesticides from other sources. Formulated acetamiprid (Mospilan^®^ 20SP a.i 20%; Target, Kartoszyno, Poland), glyphosate (Agrosar^®^ 360 SL a.i 36%; CIECH Sarzyna, Nowa Sarzyna, Poland) and tebuconazole (Tebu^®^ EW, a.i 25,8%; HELM, Hamburg, Germany) were used. Colonies were exposed daily to 0.5L for 7 days by feeding a sugar syrup Apikand Premium *(Lyson*, *Sulkowice*, *Poland)* containing formulated acetamiprid, glyphosate, and tebuconazole. Pesticides were administered alone, in binary or ternary mixtures, making 7 experimental groups. Each group consisted of 5 colonies (S1 Graphical). The names of the groups are the first letters of the active substances added to the syrup. For example, the group feeding the syrup with the addition of acetamiprid and glyphosate was called A+G. The control group was marked with the letter “C”. Feed was administered in feeders placed on the top of the hive. Maximum doses of active substances allowed for use in oilseed rape cultivation given by the pesticide manufacturer were lowered to obtain the concentration found in honey, nectar and pollen (Table 1). Range of doses found in the field was large and we rejected extreme values. Thus, the feed contained 250 ppb of acetamiprid, 7200 ppb of glyphosate, and 147 ppb of tebuconazole. Control group was fed sugar syrup Apikand Premium *(Lyson*, *Sulkowice*, *Poland)* without pesticides.

**Table 1:** Concentration of active substances found in pollen, nectar or honey and concentration used in the study.

Active substance	Formulation trade name	Literature data about the active substances detected in pollen, honey or nectar [ppb]	Concentration of the active substances used in the study [ppb]
**Acetamiprid**	Mospilan 20 SP	0,002–14 800 [40,43,82–85]	250
**Glyphosate**	Agrosar 360 SL	20–120 000 [12,13,38,41,86,87]	7 200
**Tebuconazole**	Spekfree 430 S.C.	0,12–4 530 [17,39,41,42,45,46,83,85]	147

### Biochemical analysis

To collect the hemolymph, the bee was placed on a wooden stand. After removing the antennae, the abdomen was gently pressed (from back to front) to allow hemolymph to flow out. Hemolymph was collected in end-to-end glass capillaries with a capacity of 20 μl without anticoagulant. Capillaries (10 pieces) were placed in a 2 ml Eppendorf tube with 200 μl of distilled water. There were 6 pulled samples of 10 capillaries for each group. To avoid hemolymph melanization, the entire process was performed on cooling blocks, after collecting a tube (i.e., 10 pieces of capillaries) the material was placed in a freezer (-25°C) and then at -80°C until analysis [88].

Activity of enzymes and content of non-enzymatic antioxidants were performed using a Pentra 400 biochemical analyzer (HORIBA ABX Diagnostics, France) with the use of original manufacturer’s kits Total antioxidant status was determined using a ready to use kit from Randox Laboratories Ltd., Great Britain.

#### Alanine aminotransferase (ALT), aspartate aminotransferase (AST), alkaline phosphatase (ALP)

The reagent composition for AST, ALT and ALP was as following [89]:

AST: 2-Ketoglutarate (13 mmol/L), L Aspartate (220 mmol/L), LDH (1200 U/L), MDH (90 U/L), NADH (10 mmol/L), Tris buffer (88 mmol/L) and EDTA (5.0 mmol/L), pH 8.1
ALT: 2-ketoglutarate (13 mmol/L), L-alanine (440 mmol/L), NADH (0.10 mmol/L), LDH (1800 U/L), Tris buffer (97 mmol/L) and EDTA (50 mmol/L), pH 7.8
ALP: 2-amino-2-methyl-1-propanol (900 mmol / L), magnesium acetate (1.6 mmol / L), zinc sulfate (0.4 mmol / L) and HEDTA (2, 0 mmol / L).

For AST or ALT activity measurement 100 μl of appropriate reagent solution was mixed with 10 μl of hemolymph, vortexed for 3–5 seconds and heated at 37°C for 30 seconds. The absorbance was measured at four time points (0, 1, 2 and 3 minutes) after incubation at 340 nm.

For ALP activity measurement, 100 μl of the reagent was mixed with 2 μl of hemolymph, vortexed for 3–5 seconds and heated at 37°C for 60 seconds. Then 20 μl of 4-NPP (16.0 mmol/L) was added and reaction solution was vortexed for 3–5 seconds and heated at 37°C for 60 seconds. The absorbance was measured at four time points (0, 1, 2 and 3 minutes) after incubation at 405 nm For AST or ALT activity measurement, 100 μl of appropriate reagent solution was mixed with 10 ul of the hemolymph, vortexed for 3–5 seconds and heated at 37°C for 30 seconds. The absorbance was measured at four time points (0, 1, 2 and 3 minutes) after incubation at 340 nm [75,89].

The absorbance values must be entered into the formula to calculate ALT, AST and ALP activity [75]:

$$ALT\;\text{activity}=\frac{\Delta \text{abs}}{\text{min}}\times FACTOR\;[U/l]$$

$$A\text{S}T\;\text{activity}=\frac{\Delta \text{abs}}{\text{min}}\times FACTOR\;[U/l]$$

$$AL\text{P}\;\text{activity}=\frac{\Delta \text{abs}}{\text{min}}\times FACTOR\;[U/l]$$

$$ALT\text{/}AST\;\text{FACTOR}=\frac{\text{TV}\times 1000}{6.3\times \text{SV}\times \text{P}}$$

$$ALP\;\text{FACTOR}=\frac{\text{TV}\times 1000}{18.8\times \text{SV}\times \text{P}}$$

$$\frac{\Delta \text{abs}}{\text{min}}=\frac{\left(\text{A}2-\text{A}1)+(\text{A}3-\text{A}2)+(\text{A}4-\text{A}3\right)}{3}$$

where:

A1, A2, A3, A4—individual readings of the absorbance values for the samples

TV—total volume of the reaction mixture

SV—sample volume used for the reaction

P—optical path length of the cuvette

6.3—absorbance factor for dihydronicotinamide adenine dinucleotide (NADH; at 340-nm wavelength)

18.8—absorbance factor for 2,4-dinitrophenol (2,4-DNP)

#### Gamma-glutamyl transpeptidase (GGTP)

A GGTP assay kit (ABX Pentra GGT CP, HORIBA ABX Diagnostics, France) was used to measure GGTP activity. In each analysis, 10 μl of hemolymph were used. Theproduct concentration was determined colorimetrically by measuring the absorbance at a wavelength of λ = 405–410 nm.

The level of non-enzymatic antioxidants was measured using kits from HORIBA ABX Diagnostics, France. The volume of hemolymph taken for analysis is indicated in parenthesis. Analyses were conducted according to the manufacturer’s instructions [89]:

albumin: ABX Pentra Albumin CP (2 μl)
creatinine: ABX Pentra Enzymatic Creatinine CP (10 μl)
uric acid: ABX Pentra Uric Acid CP (5 μl)
urea: ABX Pentra Urea CP (3 μl)

#### [75] Total antioxidant status (TAS)

The colorimetric method assessed the TAS of hemolymph. ABTS^®^, after incubation with metmyoglobin and H_2_O_2_, turns into the blue-green cation radical ABTSo+, whose absorption was measured at 600 nm.

$$HX\text{-}FeIII+H_{2}O_{2}\to X\;\text{-}\;\left[FeIV=0\right]+H_{2}O$$

$$ABTS^{®}+X\;\text{-}\;\left[FeIV=0\right]\to ABTS^{®}++HX\text{-}FeIII$$

HX-FeIII = Metmyoglobin

X—[FeIV = 0] = Ferrylmyoglobin

ABTS^®^ = Diammonium 2,2’-Azino-di-[3-ethylbenzothiazoline-6-sulfonate]

Calculations:

$$\text{Factor}=\frac{\text{standard}\;\text{concentration}}{\Delta \text{A}\;\text{blank}-\Delta \text{A}\;\text{standard}}$$

Where ΔA = A2 − A1

A1—initial absorbance value

A2—absorbance value after 3 minutes of reaction.

Based on the factor values, the total antioxidant potential (TAS) was calculated: TAS = factor × (ΔA blank—ΔA serum).

### Statistical analysis

The statistical package R Core Team (2013) was used for data analysis. The data distribution in each group was tested using Shapiro-Wilk test. The statistical significance of data within and between groups was first determined by the non-parametric Kruskal Wallis test with Holm correction for multiple comparison using the package “agricolae” for “kruskal” function. To perform post-hoc analysis, Dunn’s test with Bonferroni correction was used. For all tests, RStudio and a significance level of α = 0.05 were used.

### Residues analysis

The analysis of pesticides residues was carried out for sugar syrup and sugar cake, to eliminate risk of contamination of feed by plant protection products. For analysis of acetamiprid and tebuconazole residues, the PN-EN 15662 (QuEChERS) method was used using an analysis based on liquid chromatography with tandem mass spectrometry (LC-MS/MS) after partitioning with acetonitrile and purification by dispersive solid phase extraction (SPE). This analysis was performed with Agilent Technologies 1200 Series liquid chromatograph equipped with a 6410 Triple Quad LC/MS mass detector, separation on an Eclipse Plus C18 column, 100 mm × 2.1 mm, 1.8 μm. Glyphosate residues were determined by the EURL-SRM QuPPe method using extraction with methanol and LC-MS/MS. For this analysis was used Agilent Technologies 1260 Infinity II series liquid chromatograph equipped with a 6470A Triple Quad LC/MS mass detector, separation on a Hypercarb column 100 mm × 2.1 mm, 5 μm. The analyzed active substances from pesticide formulation were not found in sugar syrup and sugar cake. All analyses were performed by Food Safety Research Department of the Institute of Horticulture—National Research Institute in Skierniewice, Poland.

## Results

### Enzymes activity

**Fig 1:**
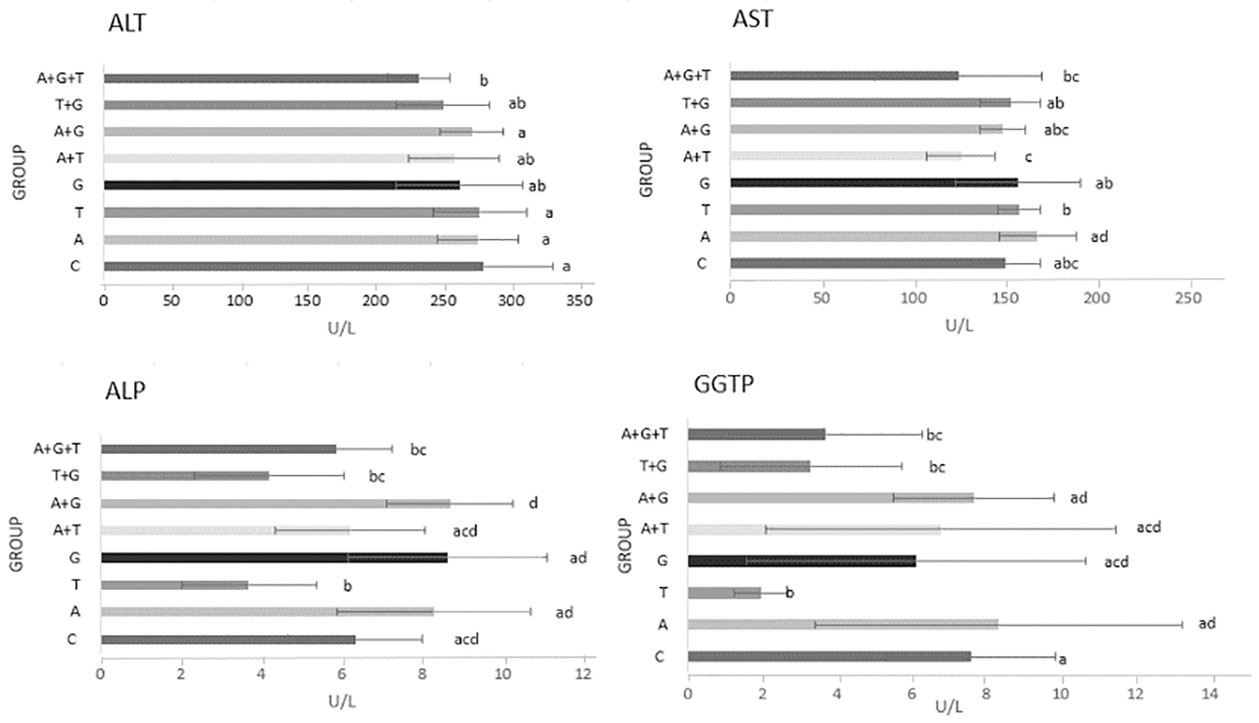
Enzymes activity in hemolymph of honey bee workers. ALT—alanine aminotransferase; AST–aspartate aminotransferase; ALP—alkaline phosphatase; GGTP—gamma-glutamyl transpeptidase. Bars demonstrate mean value, errorbars present standard deviation, lowercase letters on the right show the result of the Kruskal-Wallis test with Holm correction for multiple comparisons, α = 0.05 (groups with the same letter do not differ significantly). Control (C) was fed with clear sugar syrup, other groups syrups contained pesticide active substance (G–glyphosate, T–tebuconazole, A–acetamiprid).

ALT activity was lower in all experimental groups compared to the control group (Kruskal-Wallis chi-squared = 21.96, p-value = 0.002581, df = 7). In the case of bees fed with the ternary mixture (group A+G+T) activity decreased by 16.9% (Fig 1). AST activity was not statistically different between the experimental and control groups (Kruskal-Wallis chi-squared = 35.67, p-value = 8.365e-06, df = 7). Comparatively to the control group, ALP activity was significantly lower (41.6%) in the T group (Kruskal-Wallis chi-squared = 73.653, p-value = 2.692e-13, df = 7). GGTP activity in the bee hemolymph was considerably decreased in the T group compared to the control group (a decrease of 74.1%) (Kruskal-Wallis chi-squared = 50.897, p-value = 9.625e-09, df = 7). This change was also significantly different from the change caused by acetamiprid (group A) and the combination of acetamiprid and tebuconazole (group A+T) (Fig 1). The ternary mixture also greatly affected GGTP activity, reducing it by 51.2% compared to the control group. In addition, GGTP activity significantly decreased in group T+G.

### Non-enzymatic antioxidants

**Fig 2:**
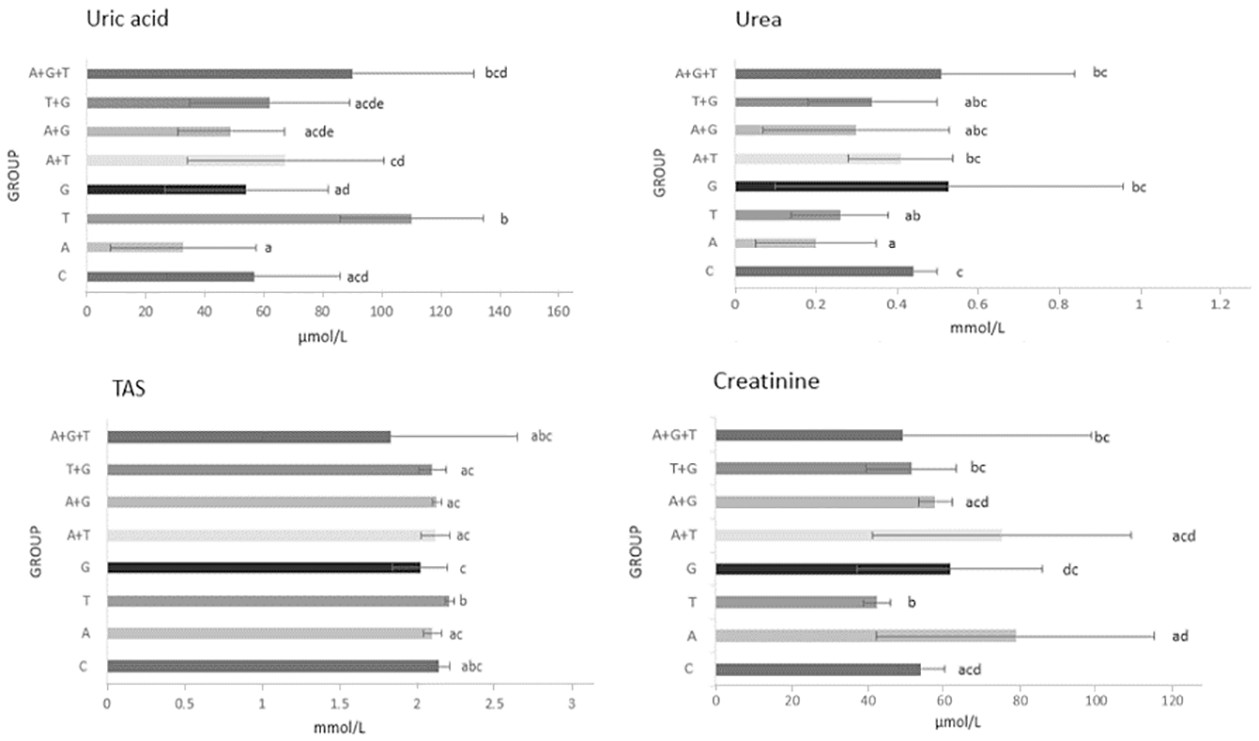
Non-enzymatic antioxidants in hemolymph of honey bee workers. TAS–Total Antioxidant Status. Bars demonstrate mean value, errorbars present standard deviation, lowercase letters on the right show the result of the Kruskal-Wallis test with Holm correction for multiple comparisons, α = 0.05 (groups with the same letter do not differ significantly). Control (C) was fed with clear sugar syrup, other groups syrups contained pesticide active substance (G–glyphosate, T–tebuconazole, A–acetamiprid).

For single substances, acetamiprid and tebuconazole led to a statistically significant decrease in urea levels compared to the control group (by 53.6% and 40.9%, respectively) (Kruskal-Wallis chi-squared = 34.012, p-value = 1.713e-05, df = 7) (Fig 2). Tebuconazole also caused a significant decrease in creatinine levels (by 21.5%) (Kruskal-Wallis chi-squared = 52.191, p-value = 5.354e-09, df = 7), and an increase in uric acid levels (by 94.8%) (Kruskal-Wallis chi-squared = 52.651, p-value = 4.344e-09, df = 7) in comparison to the control group. Glyphosate did not significantly affect the level of any of the analyzed markers.

### TAS

There were no significant differences in the TAS level between the experimental groups and the control group (Fig 2) (Kruskal-Wallis chi-squared = 37.754, p-value = 3.374e-06, df = 7). Bees receiving syrup with the addition of tebuconazole were characterized by statistically significantly higher levels of TAS than bees from the groups receiving syrup with the addition of glyphosate, acetamiprid and binary mixtures.

## Discussion

During this study, workers developed in the hive and were exposed to PPPs (plant protection products, i.e acetamiprid, tebuconazole and/or glyphosate) mixtures in concentrations that corresponded to residues found in pollen, honey, and/or nectar. It was our goal to determine if residues of plant protection products had a negative effect on biochemical parameters in honey bee workers.

Insecticide toxicity can be increased by triazole fungicides [28,29,32,90]. In limited studies, interactions between pesticide-active substances have been examined; however, they indicate different effects when they are combined [14,37,52,53,91–94]. Individual PPPs’ active substances may interact, changing their toxicity to honey bees [22]. A lot of studies on pesticides and their toxicity to honey bees are using active substances in the simplest possible formulation (i.e. with one solvent). It is worth emphasizing that commercial formulations are more complex and additives can be toxic to bees or change the toxicity of active substances [36,94]. Our findings show that biochemical markers were not affected by pesticide mixtures in a greater or equal way than the combined effects of two separate substances. This means that the tested active substances show an antagonistic effect when combined. Interestingly, our previous studies on these mixtures showed that the same formulations as in current study, can have synergistic effect on workers mortality [37,91]. There is evidence that different concentrations of substances in mixtures can cause different effects [36]. Binary mixtures did not cause statistically significant changes in the activity of any of the studied markers in the bee hemolymph, except the T+G group and GGTP activity. However, in many cases, the changes induced by single agents differed significantly from the changes induced by their combination. Changes in the activity of the analyzed enzymes caused by the ternary mixture were significantly different from changes caused by at least one active substance in the case of all enzymes, except for ALP. Active substances, depending on the combination in the mixture, had a different effect on the level of the analyzed indicators. Glyphosate in a mixture with acetamiprid increased ALP and GGTP activity, and in combination with tebuconazole decreased their activity. Tebuconazole in combination with acetamiprid decreased AST activity and increased its activity in combination with glyphosate.

Pesticides can change detoxification and antioxidant mechanisms and affect the whole organism of the honey bee. Many studies have confirmed that pesticides altering enzyme activity and change the level of some key substances (e.g. ATP, proteins and glutathione) [14,50,52,58].

In response to harmful substances, the parasitic activity of *Varroa destructor* [56,57], and electromagnetic fields [74], ALT, AST, and ALP activity in bee hemolymph decreases. Alternatively, it can increase in response to stress (e.g., high temperatures [62] and stimulating substances, such as curcumin [69,70,95]). The effects of imidacloprid, a neonicotinoid insecticide, on honey bee colonies were investigated by Paleolog et al. (2020) [51]. This pesticide reduced ALP, ALT, and AST activities. In our study ALT activity was significantly lower in A+G+T group compared to the control group, while AST activity was not statistically different between the experimental and control groups. ALP activity was significantly lower in the T group (Fig 1).

Only few studies have examined GGTP activity in honey bee hemolymph. GGTP activity can be increased in honey bee hemolymph as a result of stress factors, like high temperatures [62]. Veterinary drugs also decreased the level of this enzyme sug [61]. In our study GGTP activity in the bee hemolymph was considerably decreased in the T, T+G and A+T+G group (Fig 1).

In our research, acetamiprid at 250 ppb administered alone to bee colonies did not cause significant changes in the activity of the tested enzymes and in the level of most non-enzymatic antioxidants (Figs 1 and 2). The exception was the urea level, which was significantly lower than the control group (by 54.5%). The study of Migdal et al. showed that acetamiprid increased AST and ALT activity and decreased creatinine level in hemolymph at higher concentrations (200,000 ppb) [91]. Further, this insecticide (600, 1200, 2400, 6000 and 60,000 ppb) affected also polyphenol oxidase and carboxylesterase, and detoxification enzyme GST [21]. Urea levels change under veterinary drugs and stimulants. In the research by Strachecka et al. urea levels in bees exposed to bromfenvinphos decreased significantly (three times) in the hemolymph of bees over 7 days of age [57]. In bees under the influence of stress related to too high temperature, urea levels increased [62].

Our study shows that glyphosate at 7200 ppb did not significantly affect the activity of any of the analyzed indicators (Figs 1 and 2). Similar conclusions were reached in study of Migdal et. al. in the acute toxicity test (20000000 ppb) [91]. In the studies of Helmer et al. bees receiving syrup with glyphosate (1.25, 2.50 and 5.0 ng/bee) were characterized by a reduced content of beta-carotene, which has an antioxidant effect [96]. In other studies, depending on the concentration, method of application and age of the bees, this herbicide may affect the level of antioxidant and detoxification indicators of the honey bee [54,97].

In our present research, tebuconazole administered to bees at 147 ppb reduced the activity of GGTP and ALP enzymes compared to the control group. Further, it caused an almost two-fold increase in the uric acid concentration in the hemolymph of honey bee workers and lowered the creatinine and urea levels compared to the control group (by 21.5% and 40.9%, respectively). The effect of fungicides, including triazole fungicides, on the level of physiological indicators of honey bees is an issue that has not yet been investigated well [98]. In acute toxicity test (625000 ppb) tebuconazole increased ALP activity in hemolymph [91]. Although triazole fungicides may increase insecticide toxicity to the honey bee [27,29,32], our research did not confirm this effect.

Maintaining bee colonies for experimental purposes in controlled conditions (i.e. having an influence on the weather and having complete control over the feeding place and the place where the bees fly is impossible [80]. We decided to feed bees in the hive to be sure that they were exposed to tested pesticides. Unlike in laboratory research, we cannot assume what exact doses of pesticide are consumed by individual worker bees. Bees can store food and consume it at different times. The way the pesticide was distributed in a bee colony and the interactions that occurred between various substances in the hive were also unpredictable. However, it is an important aspect to investigate how pesticides affect bee workers in the hive. In laboratory conditions it is not possible to create a scenario where workers store food in the hive, process it, manage and share it with other bees. The next step is to investigate how mixtures of pesticides used in the field affect colonies in field research.

## Conclusion

The highest number of differences was observed among bees fed syrup containing tebuconazole (group T). Five of the nine biochemical markers were significantly different from the control group (GGTP, ALP, creatinine, uric acid, and urea). Each of them was lower than the control, except for uric acid, which was 98% higher (Figs 1 and 2). In the case of bees in groups G, A+T, and A+G the level of any of the analyzed markers did not differ significantly from the control group. Pesticides and their mixtures in concentrations corresponding to residues detected in pollen, honey, and/or nectar cause significant changes in the level/activity of some of the examined biochemical markers of worker bees hemolymph. Pesticides used individually and in mixtures had a different effect on biochemical markers. In each case, tested active substances show an antagonistic effect when combined. Administered alone, acetamiprid (insecticide) significantly decreased the level of urea in the hemolymph of worker bees. Glyphosate (herbicide) did not affect significantly the level/activity of any of the analyzed biochemical indicators of worker bees. Tebuconazole (fungicide) caused changes in the levels of most of the studied biochemical markers. We found that tebuconazole, which as a fungicide is generally considered safe for bees, may be harmful and more research is required. The impact of fungicides is a crucial element of the assessment of threats to the honey bees.

## Supporting information

S1 Graphical (TIF)
