# Supplementary figures and images for: Semi-field studies on biochemical markers of honey bee workers (Apis mellifera) after exposure to pesticides and their mixtures
Source: PLoS One. 2025 Jan 30;20(1):e0309567. doi: 10.1371/journal.pone.0309567 (PMC11781695; doi:10.1371/journal.pone.0309567)

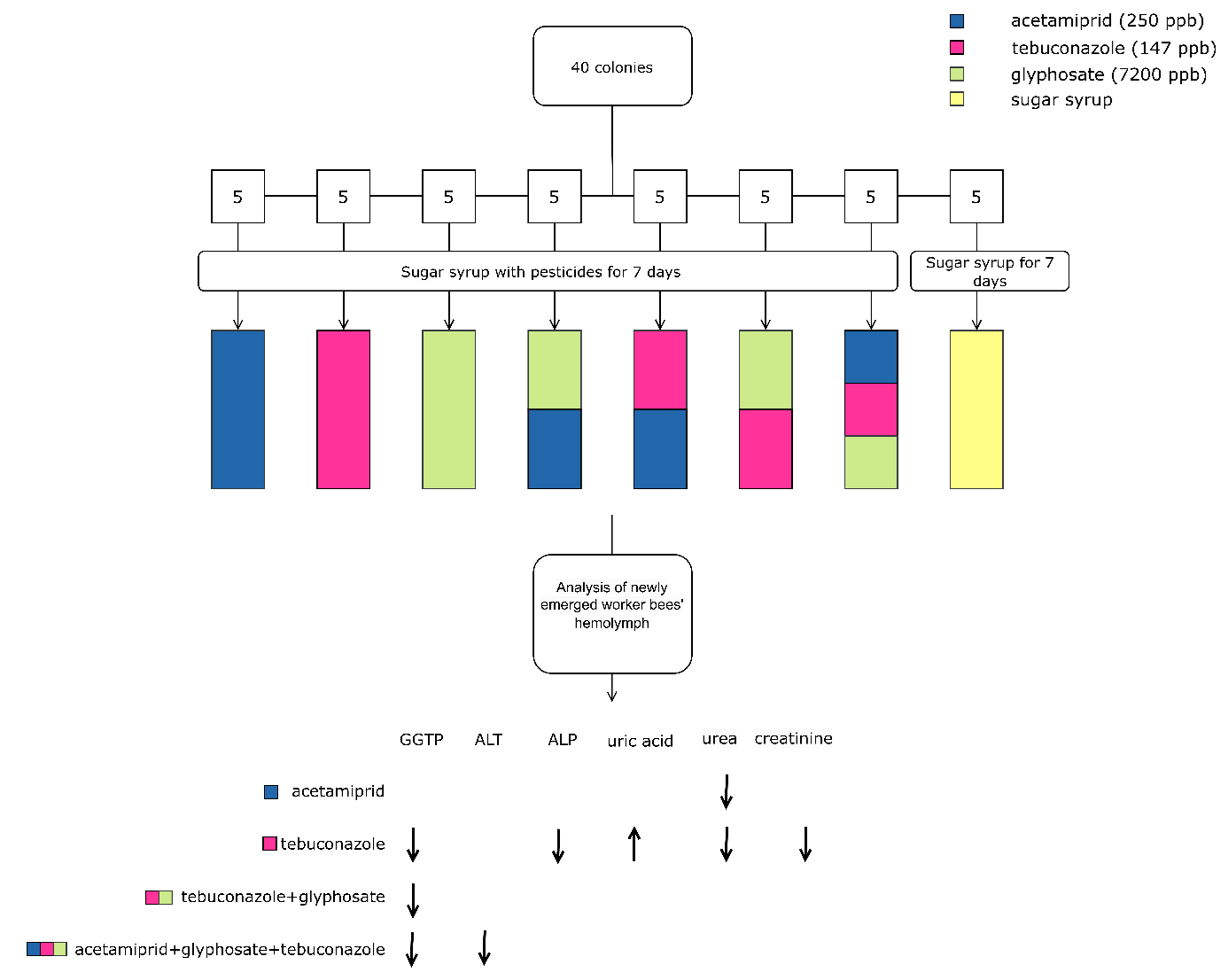

Supplement: S1 Graphical — (TIF) [file pone.0309567.s001.tif]
